# Digital infrastructures and data interoperability in radiology for prevention: from digital health to AI-enabled, quantitative imaging workflows

**DOI:** 10.1007/s11547-026-02263-4

**Published:** 2026-07-13

**Authors:** Luca Maria Sconfienza

**Affiliations:** 1 Università degli Studi di Milano, Milan, Italy; 2 IRCCS Istituto Ortopedico Galeazzi, Via Cristina Belgioioso 173, 20157 Milan, Italy

**Keywords:** Artificial intelligence, Data interoperability, Digital infrastructures, Radiology

## Abstract

Radiology is rapidly evolving from a service that produces images into a data-centric clinical platform that supports prevention, early diagnosis, and personalized care. This shift is accelerated by the convergence of digital health, artificial intelligence (AI), and quantitative imaging approaches such as radiomics. However, the clinical impact of these innovations depends less on algorithms alone and more on robust digital infrastructures and true interoperability across radiological, clinical, and—when available—molecular data. In practice, many implementations fail because data remain fragmented across heterogeneous systems, metadata are incomplete, workflows are not harmonized, and governance frameworks are insufficient to ensure quality, privacy, and accountability. This paper focuses on the radiology-centric requirements for interoperable data ecosystems, covering technical and semantic standards (e.g., DICOM, HL7 FHIR, IHE profiles, controlled terminologies), data governance and quality programs, and AI integration into real-world radiology workflows. Common practical barriers, including legacy IT debt, semantic inconsistencies, model drift, bias, medico-legal uncertainty, and hidden operational costs, are discussed. Last, pragmatic recommendations to enable scalable, secure, and clinically useful interoperability are proposed—supporting prevention pathways and the responsible deployment of AI and radiomics at institutional and network levels.

## Introduction: radiology as a prevention-grade data platform

Modern prevention starts depending on data that are longitudinal, comparable, and actionable. Radiology is located in the very center of this transformation. Imaging contributes to screening, risk stratification, early detection, and follow-up and increasingly produces quantitative outputs (measurements, volumetry, densities, scores, and radiomics signatures). Yet the value of imaging data is often constrained by how it is stored, labeled, shared, and interpreted across systems.

In most institutions, imaging is still treated primarily as visual content, managed in picture archive and communication systems (PACS) that were designed for retrieval and display rather than for computable reuse. Meanwhile, prevention-oriented care requires that imaging data can be linked to clinical context (indication, symptoms, laboratory values, therapies), outcomes (follow-up imaging, pathology, adverse events), and—where available—molecular or genomic features. This cannot be achieved without interoperable infrastructures that ensure data integrity, semantics, and governance.

Crucially, the success of AI and radiomics is not determined only by model performance in controlled settings, but by the reliability of data flows and the “fit” of these tools within daily workflow. If AI output arrives late, in a separate portal, with unclear meaning or poor traceability, it becomes an additional burden rather than a clinical tool. Therefore, infrastructures and interoperability are not supporting details; they are the primary determinants of real-world impact.

Prevention-oriented workflows impose additional constraints that amplify interoperability gaps: They often operate in low-prevalence settings where small changes in specificity can generate substantial downstream burden, while small decreases in sensitivity may delay diagnosis. Prevention also depends on longitudinal decisions (interval change and follow-up adherence) over years, making protocol and metadata consistency essential. Finally, many prevention programs scale across sites, increasing the need for organizational interoperability, governance, and auditable tracking of recommendations and outcomes.

This manuscript is a practical position paper intended to provide a radiology-centric framework and actionable recommendations for interoperable digital infrastructures supporting prevention-oriented workflows. Recommendations were developed through a targeted narrative literature search covering interoperability standards and profiles (DICOM/DICOMweb, HL7 FHIR, IHE), controlled terminologies and structured reporting, AI reporting/deployment guidance (including post-deployment monitoring), and radiomics reproducibility/standardization frameworks, combined with the authors’ expert synthesis of recurrent implementation failure modes and feasible mitigations. No formal Delphi process or systematic evidence grading was performed; accordingly, statements should be interpreted as pragmatic best practices rather than graded guideline recommendations.

## What interoperability means in radiology: three complementary domains

Interoperability in healthcare is often discussed as simple connectivity. However, in radiology, it concerns three different domains:

### Technical interoperability

Systems must exchange information reliably through well-defined standards and interfaces. In radiology, this includes image and metadata exchange and the linkage between ordering, scheduling, acquisition, storage, reporting, and distribution. DICOM provides the foundational standard for medical images and related information, ensuring that imaging objects can be shared with the fidelity needed for clinical use [1].

Practical limitation: Institutions frequently experience “interoperability by export,” relying on ad hoc formats (e.g., PDFs, manual downloads and uploads, proprietary viewers). This creates fragile workflows, delays, and downstream data loss and the impossibility of quickly integrating external platforms. Mitigation/impact: Prioritize standard interfaces (DICOM plus DICOMweb where available) and enterprise ingestion workflows with metadata preservation; if unaddressed, the impact is high in prevention due to scale and longitudinal follow-up needs.

### Semantic interoperability

Even when systems can exchange data, their meaning must be consistent. For radiology, semantics include standardized procedure naming, consistent anatomical/pathological labeling, uniform metadata capture, and computable reporting elements. Controlled vocabularies (e.g., RadLex, SNOMED CT) and structured reporting frameworks enable comparable datasets and improve downstream analytics [2, 3].

Practical limitation: Semantic misalignment is the most common hidden failure mode. “The data move, but they cannot be compared/understood.” This is particularly damaging for radiomics and AI, where subtle differences in acquisition or labeling can compromise reproducibility. Mitigation/impact: Define a minimum semantic rule set (laterality, anatomy, indication), bind to controlled terminologies, and run periodic semantic audits; if unaddressed, the impact is high for longitudinal tracking, AI generalization, and radiomics reproducibility.

### Organizational interoperability

Workflow roles, responsibilities, and timing must be integrated across departments and sites. Integrating the Healthcare Enterprise (IHE) profiles formalize interoperable radiology workflows (e.g., Scheduled Workflow) by defining how registration, order communication, acquisition, and storage should interoperate in clinical practice [4].

Practical limitation: Local organizational variability (different ordering processes, inconsistent patient identifiers, divergent imaging protocols) can break interoperability even when standards exist. Mitigation/impact: Establish cross-site governance for patient identity, procedure catalogs, and protocol versioning, with end-to-end integration testing; if unaddressed, the impact is high for multi-site prevention pathways.

## Digital health and the radiology data ecosystem: from PACS to reusable data

A prevention-grade radiology ecosystem requires more than a PACS. It requires an architecture where imaging and clinical information are consistently linked and retrievable for care and secondary uses.

### Core components

A typical ecosystem includes:

- Radiology information system (RIS) for scheduling, worklists, reporting, and operational tracking
- PACS/VNA for imaging archive continuity and enterprise distribution
- Diagnostic viewer with quantitative tools and AI integration points
- Electronic health record (EHR) integration for clinical context (laboratory, notes, medications, risk factors)
- Digital document repositories for consent, reports, and structured outputs
- Integration layer (standards-based interfaces/APIs)
- Data platforms (warehouse/lake) for analytics, quality assurance, radiomics pipelines, and AI monitoring

### The central role of metadata

In radiology, images without metadata are “incomplete clinical objects.” Metadata are essential for: (i) comparing exams over time, (ii) enabling automated quality checks, (iii) supporting radiomics reproducibility, and (iv) detecting workflow mismatches (wrong patient, wrong side, incomplete protocol). DICOM defines not only image formats, but related information structures needed for clinical exchange [1].

Practical limitation: Metadata completeness is frequently suboptimal—especially when examinations are imported from external sources, performed in emergency settings, or stored in systems that do not enforce consistent tagging. Mitigation/impact: Enforce minimum metadata capture at ingestion, implement protocol naming/versioning, and deploy automated QC checks; if unaddressed, the impact is high for longitudinal comparability and quantitative pipelines.

### Data governance and data quality are non-negotiable

Data governance establishes rules for ownership, access, accountability, retention, and quality. In practice, the most damaging barrier to AI adoption is not compute capacity but poor data quality: inconsistent procedure naming, missing indications, wrong laterality, duplicated patients, or narrative-only reports. Without a structured data-quality program (metrics, audits, corrective workflows), AI may simply accelerate the production of erroneous outputs. In practice, “garbage in, garbage out” remains the main cause of failure. To move from principles to implementation, radiology departments benefit from defining a pragmatic “minimum interoperability dataset” that must be available and linkable across RIS/PACS/EHR and downstream analytics environments. This minimum dataset should include not only identifiers and clinical context but also acquisition and reconstruction parameters that are essential for quantitative imaging, radiomics reproducibility, and longitudinal comparability. Table 1 shows a potential radiology-centric minimum interoperability dataset designed to support AI integration, registries, and radiomics-ready workflows.

**Table 1:** Minimum interoperability dataset for radiology AI, registries, and radiomics-ready workflows (radiology-centric)

Domain	Minimum elements	Why it matters (practical rationale)
Patient identity and linkage	Unique patient ID; encounter/episode ID; site/facility ID; consent/secondary-use status (where applicable)	Prevents mismatches; enables longitudinal follow-up and cross-system linkage; supports lawful governance for secondary use
Order and indication	Referring unit; clinical question/indication (coded where possible); priority/urgency; relevant risk factors (screening flags)	Essential context for prevention pathways, triage, and appropriate AI routing; reduces spurious comparisons and improves interpretability
Study identifiers	Accession number; study date/time; modality; body region; laterality; protocol name + version	Enables reliable retrieval, comparability, and correct grouping of exams for analytics/AI; protocol versioning is critical for drift detection
Acquisition parameters (core)	Vendor/model; field strength (MR)/kVp-mAs (CT)/frequency and presets (US); slice thickness; spacing; reconstruction kernel/algorithm; contrast use (yes/no + timing); dose indicators (CTDIvol/DLP where relevant)	Radiomics and quantitative analytics are highly sensitive to acquisition/reconstruction. These fields are minimal to ensure reproducibility and interpret model performance changes over time
Image series metadata	Series description; anatomical coverage; phase/sequence identifiers; orientation; key series flags (e.g., diagnostic series vs localizers)	Ensures correct AI/radiomics series selection; supports automation and QC (avoids processing wrong series)
Quality indicators	Motion/artifact flags; completeness checks (coverage); repeat acquisition indicators	Drives QA, reduces downstream errors, and supports automated “stop” conditions when images are not suitable for AI/radiomics
Quantitative measurements	Standardized key measures (e.g., lesion size/volume, density, scores) with units and method; longitudinal deltas where applicable	Enables prevention-oriented tracking; supports structured reporting and outcome analytics; reduces variability and manual transcription
Annotations/segmentation (if used)	ROI/segmentation objects; annotation provenance (who/when/tool); versioning; inter-reader agreement where relevant	Critical for radiomics and training/validation datasets; provenance and versioning prevent “annotation drift” and enable auditability
Structured report elements	Key findings (coded where possible); BI-RADS/PI-RADS/Lung-RADS or analogous scales; follow-up recommendation fields; critical results flags	Converts narrative into computable data; supports pathway adherence, registry integration, and automated follow-up management
Downstream outcomes	Pathology results (where applicable); treatment start; follow-up imaging outcome; adverse events; survival endpoints (research)	Allows real-world validation of AI/radiomics; enables prevention KPIs and longitudinal effectiveness tracking
Operational workflow metadata	Report timestamps (start/final); reader role; addenda; discrepancy/peer review outcomes	Supports workflow analytics, turnaround metrics, and quality improvement; essential to quantify operational value of AI integration
AI-specific traceability (if deployed)	AI tool name/version; input series IDs; output type (triage/measurement/alert); confidence/uncertainty; user interaction log	Enables auditing, medico-legal defensibility, drift tracking, and safety monitoring in real-world use

## Standards and practical interoperability: DICOM, HL7, fast healthcare interoperability resources (FHIR), and integrating healthcare enterprise (IHE) profiles

### DICOM for imaging exchange

DICOM is the international standard that defines formats for medical images and related information, supporting exchange with the quality required for clinical use [1]. It is foundational for imaging interoperability and remains the backbone for modality-to-archive and archive-to-viewer communication. Although created in 1985, well ahead of the introduction of AI in clinical practice, DICOM still represents a very robust standard.

### HL7 FHIR for modern, API-centric clinical data exchange

HL7 FHIR is a widely adopted standard for exchanging healthcare information electronically, designed with modern web technologies and API-focused implementations [5]. For radiology, FHIR enables closer linkage between imaging events and clinical data, supporting downstream decision support, longitudinal tracking, and cross-system integration.

In practical radiology deployments, FHIR adds value when used to (i) represent imaging orders and indications (e.g., as ServiceRequest), (ii) link performed studies to the clinical episode (e.g., ImagingStudy), and (iii) deliver reports and key structured findings back to the EHR (e.g., DiagnosticReport and coded Observations). For prevention pathways, this enables longitudinal tracking of follow-up recommendations and auditable pathway adherence at population scale.

A common implementation pattern is to keep DICOM as the source of truth for imaging objects while using FHIR resources to orchestrate clinical context, patient/episode linkage, and retrieval pointers (often via DICOMweb). Key limitations include profile alignment, terminology binding, and identifier consistency across systems; these should be addressed with institutional implementation guides and end-to-end testing.

Practical limitation: “FHIR availability” does not automatically ensure meaningful interoperability. Institutions must still align resource profiles, terminologies, and identifiers. Additionally, imaging-specific needs may require careful bridging between DICOM objects and clinical FHIR representations. Mitigation/impact: Establish cross-site governance for patient identity, procedure catalogs, and protocol versioning, with end-to-end integration testing; if unaddressed, the impact is high for multi-site prevention pathways.

### IHE workflow profiles: where standards become operational

IHE integration profiles help to put interoperability in place by specifying concrete workflow transactions across systems. For example, Scheduled Workflow integrates ordering, scheduling, acquisition, storage, retrieval, and display for radiology exams [4]. While DICOM, HL7 FHIR, and IHE profiles define how information can be exchanged, operational success depends on aligning what information is exchanged consistently and completely. In practice, implementing standards is most effective when paired with a clearly defined minimum dataset requirement across systems, such as the elements summarized in Table 1.

Practical limitation: Many failures stem from partial implementation or inconsistent configuration across vendors. Even when a profile is supported, end-to-end validation and governance are needed to avoid “standards-compliant but functionally broken” pipelines. Mitigation/impact: Address with governance, standardization, and measurable QA processes; if unaddressed, the impact ranges from moderate to high depending on scale.

## Semantic infrastructure: procedure names, terminologies, and structured reporting

### Controlled terminology for radiology

RadLex is a comprehensive radiology lexicon intended for reporting, decision support, registries, data mining, education, and research [2]. SNOMED CT provides broad clinical terminology for consistent representation of clinical content in electronic health records [3]. These resources support semantic interoperability by aligning the language of radiology with the language of clinical systems.

Practical limitation: Terminology adoption requires discipline and tooling. If radiologists and referring clinicians cannot easily select standardized terms (because systems are not user-friendly, workflows are rushed or they are not trained to), semantic drift persists. Mitigation/impact: Define a minimum semantic rule set (laterality, anatomy, indication), bind to controlled terminologies, and run periodic semantic audits; if unaddressed, the impact is high for longitudinal tracking, AI generalization, and radiomics reproducibility.

### Structured reporting as a bridge to computable imaging

Structured reporting supports consistent data capture and enables analytics, AI training/monitoring, and prevention registries. It is particularly valuable in screening programs, oncology follow-up, and high-impact conditions where longitudinal comparability is essential.

Practical limitation: Structured reporting often fails due to perceived time burden or poor integration. To succeed, structured elements should be embedded naturally in the reporting environment and provide immediate clinical value (e.g., auto-populated measurements, guideline-driven follow-up suggestions). Mitigation/impact: Start with high-impact pathways (screening/surveillance), keep templates concise, and auto-populate quantitative fields; if unaddressed, the impact is moderate-to-high because recommendations and outcomes remain non-computable.

## AI and the radiology workflow: from model accuracy to operational value

AI delivers clinical value in radiology when it is embedded into the routine workflow—worklists, viewers, and reporting environments—so that it supports decisions at the right time without adding operational friction. In this sense, “good” radiology AI is not only accurate; it is timely, traceable, and actionable.

### Workflow-native AI use cases

- A first high-impact use case is worklist triage and prioritization, where AI helps surface time-sensitive examinations (e.g., suspected acute findings) to reduce delays in reporting.
- A second is acquisition and quality control, including automated alerts for inadequate coverage, motion artifacts, or protocol mismatches, which can prevent downstream diagnostic uncertainty and reduce repeat scans.
- Third, AI can provide automated quantification—measurements, volumetry, density metrics, or scores—reducing variability and enabling structured capture of quantitative information.
- Fourth, AI can support reporting, for example, by checking completeness, pre-filling standardized elements, or highlighting inconsistencies between indication, findings, and conclusions.
- Finally, AI can facilitate longitudinal tracking, identifying prior comparable studies and summarizing interval change, which is especially relevant for prevention pathways and surveillance programs.

These applications depend on interoperability elements that are often underestimated: reliable identifiers, consistent series metadata, protocol versioning, and auditable logging of inputs and outputs. Without workflow-native integration (single sign-on, viewer/RIS embedding, clear provenance), even strong algorithms risk becoming separate “tools” that are rarely used in daily practice. The minimum dataset items summarized in Table 1 help ensure that AI results remain traceable, interpretable, and auditable at scale.

### Monitoring, drift, and “real-world performance”

Models can degrade when scanners change, protocols evolve, populations shift, or new reconstruction techniques are adopted. Therefore, implementation requires continuous performance surveillance (including error tracking, bias monitoring, and drift detection) and training.

Practical limitation: Many institutions deploy AI without a sustainable monitoring framework. Without real-world audit loops, models may remain in use despite silent performance degradation. Mitigation/impact: Implement post-deployment monitoring (drift indicators, periodic re-validation, subgroup checks) with ownership and “stop rules”; if unaddressed, the impact is high due to silent degradation and safety risk.

### Reporting and evidence standards for AI tools

To support trustworthy clinical adoption, AI studies and implementations should follow robust reporting frameworks. The CONSORT-AI extension provides reporting guidance for clinical trials involving AI interventions [6], and SPIRIT-AI addresses AI-specific needs in trial protocols [7]. For prediction models, TRIPOD + AI expands transparent reporting recommendations for models using regression or machine learning methods [8]. These frameworks help ensure that AI systems are evaluated and communicated in ways that support reproducibility, interpretability, and responsible deployment.

## Automation and quantitative imaging: improving precision without adding burden

Automation in radiology is often introduced to manage increasing volumes, but its most clinically meaningful contribution is the reduction of avoidable variability—differences driven by workflow, protocol selection, measurements, and reporting habits rather than by true biology. This is particularly relevant in prevention-oriented imaging, where the goal is to detect subtle changes early and to track them reliably over time.

### Where automation improves precision

- A first area is protocol standardization. Automated protocol selection and standardized protocol templates, linked to the clinical indication and pathway, can reduce heterogeneity across scanners, sites, and operators, improving longitudinal comparability. Protocol versioning is equally important: When acquisition or reconstruction settings change, documenting those changes allows clinicians and analytics/AI pipelines to interpret follow-up differences correctly.
- A second area is measurement consistency and quantitative capture. Automated or semi-automated measurements (e.g., diameters, volumes, density metrics, scores) can reduce inter-observer variability and transcription errors, enabling structured recording of quantitative outputs. The key advantage for prevention is not only a more repeatable single measurement, but a more reliable computation of change over time (longitudinal deltas).
- Third, automation supports precision through longitudinal comparison at scale: automated retrieval of relevant priors, selection of comparable series, and clear presentation of trends reduce the risk of omissions in busy clinical settings.
- Finally, decision support and completeness checks—such as prompts aligned with pathway recommendations and verification that key structured fields are present—can reduce reporting variability and improve computable reuse.

These benefits depend on upstream standardization: Automating an inconsistent process can produce outputs that are consistent but not clinically correct. For this reason, automation should be implemented alongside protocol governance, structured data capture, and continuous quality monitoring.

Practical limitation: Automation can fail if upstream processes are not standardized. Automating inconsistent workflows can increase rigid errors—precise outputs that are not clinically correct. Mitigation/impact: Address with governance, standardization, and measurable QA processes; if unaddressed, the impact ranges from moderate to high depending on scale.

## Radiomics and prevention: reproducibility depends on infrastructure

Radiomics aims to extract high-throughput quantitative features from medical images, but its clinical translation is limited by reproducibility challenges. The Image Biomarker Standardization Initiative (IBSI) provides a standardized framework for radiomics feature definitions and software verification, addressing a core barrier to reproducible quantitative imaging biomarkers [9].

Importantly, radiomics clinical translation remains fragile: Feature values can shift with reconstruction settings, scanner upgrades, segmentation variability, and differences in patient mix. Even within a single institution, achieving the degree of protocol standardization needed for robust multi-year longitudinal comparisons can be challenging. Therefore, expectations should be realistic: Radiomics is most credible when deployed in narrow-scope, protocol-governed pathways with transparent validation and ongoing quality control, rather than as a generic “plug-in” across heterogeneous imaging.

### Infrastructure prerequisites for radiomics

- A first prerequisite is harmonized acquisition and reconstruction, including consistent protocol naming and explicit protocol versioning across scanners and sites. Even small changes in slice thickness, reconstruction kernels/algorithms, denoising, or contrast timing can affect feature values and compromise comparability across time or centers.
- Second, radiomics requires complete and traceable metadata, so that feature extraction can be interpreted, quality-checked, and—when needed—adjusted for technical differences.
- Third, there must be clear standards for segmentation and annotation, including provenance (who annotated, with which tool, and which version), because segmentation variability is a major source of feature instability.
- Fourth, radiomics workflows benefit from validated, standardized feature definitions and software verification (e.g., IBSI-aligned pipelines), supporting reproducibility and facilitating multicenter collaboration.
- Finally, radiomics becomes clinically meaningful only when linked to reliable endpoints and longitudinal outcomes (follow-up imaging, pathology where applicable, treatment and clinical events). Without robust outcome linkage and governance, radiomics remains a technical exercise rather than a prevention- and decision-support tool.

In radiomics, reproducibility is highly sensitive to acquisition and reconstruction variability, making metadata completeness a first-order requirement rather than an optional technical detail. Several of the most consequential fields (e.g., slice thickness, reconstruction kernel/algorithm, contrast timing, and protocol versioning) are explicitly included in the minimum interoperability dataset proposed in Table 1, to support radiomics-ready protocol harmonization and validation.

Practical limitation: Radiomics pipelines are highly sensitive to technical variability. Without systematic harmonization and metadata governance, multicenter generalizability remains limited despite sophisticated modeling. Mitigation/impact: Enforce minimum metadata capture at ingestion, implement protocol naming/versioning, and deploy automated QC checks; if unaddressed, the impact is high for longitudinal comparability and quantitative pipelines.

## Privacy, security, and trust: making secondary use sustainable

Prevention and precision initiatives require secure secondary use of data. Within Europe, policy and regulatory frameworks emphasize secure data exchange and governance. The European Health Data Space (EHDS) regulation entered into force in March 2025 and establishes a transition toward operational, cross-border health data use with additional implementing specifications expected over time [10]. At the same time, the European AI Act entered into force in August 2024, shaping responsibilities for trustworthy AI deployment in the EU [11]. These policy trajectories reinforce the need for institutions to treat interoperability as a security and governance challenge, not only an IT project.

Under the GDPR, pseudonymization can reduce risks and support data protection, but it is not a universal solution and does not automatically render data anonymous [12, 13]. For radiology, privacy risks are amplified in multimodal datasets (imaging plus detailed clinical and genomic data), where re-identification risks can increase.

Practical limitation: Secondary use often stalls due to organizational uncertainty—who authorizes access, how permissions are audited, and how data are protected across environments. Trust requires clear governance, transparent patient communication, and robust technical safeguards. Mitigation/impact: Establish cross-site governance for patient identity, procedure catalogs, and protocol versioning, with end-to-end integration testing; if unaddressed, the impact is high for multi-site prevention pathways.

## Implementation barriers: why real-world adoption often fails

### Legacy debt and fragmented systems

Many radiology departments operate heterogeneous platforms acquired over years, creating costly, fragile integration points. Each additional connection introduces maintenance burden and cybersecurity surface.

### Semantic inconsistency

Differences in naming, labeling, and reporting undermine the comparability of imaging datasets and degrade AI performance. Table 2 summarizes common implementation pitfalls and actionable mitigations observed in real-world radiology deployments. This may help practical implementation planning.

**Table 2:** Common implementation pitfalls in radiology data interoperability and AI deployment, with potential practical mitigations

Pitfall	What happens in practice	Practical mitigations
Connectivity without meaning (technical interoperability only)	Data move across systems, but semantics are inconsistent (procedure names, laterality, anatomy labels, incomplete metadata), undermining comparability and AI performance	Adopt controlled terminologies (RadLex/SNOMED CT) for key fields; enforce mapped procedure catalogs; define a local “minimum semantic rule set” (laterality, anatomy, indication); run periodic semantic audits (e.g., top procedures and key entities)
Legacy IT fragmentation and vendor lock-in	RIS/PACS/EHR integrations are partial or brittle; manual workarounds (PDFs, external portals) cause latency, errors, and metadata loss	Implement an integration layer with documented interfaces and end-to-end testing; include interoperability deliverables in contracts (standard compliance + APIs + change control); prioritize high-yield integration paths (ordering–worklist–report–EHR)
Insufficient metadata for quantitative imaging and radiomics	Acquisition/reconstruction details are missing or not traceable, preventing reproducibility and multicenter generalization	Define and enforce a “minimum imaging metadata set” per modality/protocol family (align with Table 1); introduce protocol naming/versioning; create harmonized radiomics-ready protocols; adopt IBSI-aligned feature extraction and software verification
AI output not workflow-native	Results arrive late, in separate portals, or without traceability; users perceive added cognitive load; adoption drops	Integrate AI in worklists/viewers/RIS with single sign-on; define decision points (pre-read triage, in-read quantification, post-report follow-up triggers); provide interpretable outputs with uncertainty cues and links to supporting evidence
No post-deployment surveillance (drift and silent degradation)	Performance changes with scanners, protocols, reconstructions, or population shifts; errors accumulate unnoticed	Establish continuous monitoring (KPIs, error review, subgroup analyses, out-of-distribution flags); define re-validation checkpoints and “stop rules”; maintain version control for models and preprocessing pipelines
Bias and inequitable performance	Models underperform in certain subgroups (age, sex, comorbidity) or acquisition strata, with disproportionate impact in prevention settings	Require stratified evaluation by patient subgroups and acquisition strata; document representativeness and limitations; define governance thresholds and escalation pathways; update/retire models when drift or bias exceeds limits
Medico-legal ambiguity and unclear accountability	Uncertainty about responsibility, documentation, and incident handling reduces clinician trust and uptake	Define institutional policy: AI as decision support with clinician final responsibility; implement audit trails and incident reporting; specify documentation standards for AI-supported decisions; provide training and appropriate-use guidance
Hidden operational costs and lack of sustainability	Integration, storage, compute, cybersecurity, monitoring, and training costs are underestimated; ROI is unclear	Budget total cost of ownership (integration + MLOps + security + training + monitoring); define measurable KPIs (turnaround time, re-scan rates, errors, pathway adherence); implement phased roll-out starting from high-impact use cases
Secondary-use stalls (privacy/security uncertainty)	Research and AI development slow down due to unclear permissions, access controls, and governance for secondary use	Implement secondary-use governance (role-based access, logging, pseudonymization workflows); separate clinical and research environments with controlled pipelines; ensure transparent patient communication and documented lawful basis/consent pathways where relevant

### Hidden costs and sustainability

Beyond procurement, institutions must budget for integration, storage, compute, security, monitoring, and training. The return on investment of AI is not automatic; it must be demonstrated with measurable outcomes (workflow time, error reduction, pathway adherence, and patient benefit).

### Accountability and medico-legal uncertainty

In practice, accountability is distributed across the developer/vendor (intended use, validation, updates), the healthcare institution (local configuration, governance, training, monitoring), and the clinician (final decision-making). A defensible deployment should therefore include clear documentation of intended use and limitations, versioning of the AI system and data pipeline, auditable logs of inputs/outputs, and an incident reporting process with escalation thresholds and “stop rules” if performance degrades. These elements are particularly important in prevention pathways, where small systematic errors can propagate at population scale.

Clinicians remain responsible for decisions supported by AI. Without clear institutional policies, incident reporting mechanisms, and transparency about model limitations, adoption may be delayed due to risk concerns.

### Human factors and change management

Even high-performing tools are rejected if they disrupt workflows or increase cognitive load. Successful implementation requires training, engagement, and clear demonstration of value.

## Potential suggestions for radiology-centric interoperability

1. Start from measurable clinical and workflow needs (screening, follow-up, triage, reporting completeness).
2. Establish a data governance model with explicit ownership, access rules, and audit trails.
3. Implement continuous data-quality programs with metrics and corrective workflows.
4. Adopt standards end-to-end: DICOM for imaging objects, HL7 FHIR for clinical APIs, IHE profiles for operational workflows.
5. Commit to semantic alignment: controlled terminologies (RadLex/SNOMED CT), standardized procedure naming, and structured reporting for high-impact pathways.
6. Integrate AI into native workflow tools (worklist/viewer/RIS), avoiding parallel portals and manual operations.
7. Monitor AI post-deployment for drift, bias, and safety; establish incident management and periodic performance reviews.
8. Ensure privacy by design for secondary use, applying GDPR-aligned safeguards and transparent patient communication.
9. Enable radiomics reproducibility through acquisition harmonization and IBSI-aligned feature pipelines.
10. Invest in people and change management, building interdisciplinary teams that include radiologists, technologists, physicists, IT, clinical engineers, and data stewards.

## Conclusions

Radiology can be a central pillar of prevention and precision diagnostics, but only if imaging is treated as interoperable, high-quality, governable data—rather than as isolated visual content. Digital infrastructures and interoperability are the enabling conditions that allow AI and radiomics to deliver clinical value, reduce variability, and support longitudinal, population-scale prevention pathways. The most common implementation failures arise not from insufficient algorithmic sophistication, but from fragmented ecosystems, semantic inconsistencies, absent governance, and poor workflow integration. By adopting standards in an operationally meaningful way, investing in semantic and organizational interoperability, and implementing continuous monitoring for AI and data quality, radiology departments and networks can make prevention-oriented innovation scalable, secure, and trustworthy. The operational ingredients for success help translate interoperability from an aspirational concept into a durable clinical capability.

## Data Availability

Not applicable.
